# The Role of Extracorporeal Shock Wave Therapy in the Treatment of Muscle Injuries: A Systematic Review

**DOI:** 10.7759/cureus.44196

**Published:** 2023-08-27

**Authors:** Yuriy Mazin, Carolina Lemos, Carolina Paiva, Luís Amaral Oliveira, Andre Borges, Tiago Lopes

**Affiliations:** 1 Physical Medicine and Rehabilitation, Centro de Reabilitação do Norte, Vila Nova de Gaia, PRT; 2 Population Studies, Instituto de Ciências Biomédicas Abel Salazar, Universidade do Porto, Porto, PRT; 3 Physical Medicine and Rehabilitation, Centro de Medicina de Reabilitação da Região Centro-Rovisco Pais, Coimbra, PRT; 4 Physical Medicine and Rehabilitation, Centro Hospitalar De Trás-Os-Montes E Alto Douro, Vila Real, PRT

**Keywords:** muscular hematomas, delayed onset muscle soreness, tendinous injuries, muscle injuries, extracorporeal shock wave therapy

## Abstract

Muscle injuries commonly occur in sports and can be classified as indirect and direct, according to the 2013 Munich Consensus Statement (MCS). Since recent evidence suggests that extracorporeal shock wave therapy (ESWT) improves muscular microcirculation and may increase regeneration after acute muscle injury, we performed a systematic review following the Preferred Reporting Items for Systematic Reviews and Meta-Analyses (PRISMA) statement guidelines to access the efficacy and safety of ESWT in the treatment of patients with muscle injuries. PubMed and Cochrane were searched to screen for potentially relevant articles and the literature search was last updated in June 2023. The inclusion criteria were randomized controlled trials, observational studies, or case controls published in English, Portuguese, or Spanish that studied the effect of ESWT on indirect and direct muscle injuries in individuals aged ≥18, with at least one of the following reported outcomes: pain on the visual analog scale (VAS), functionality assessed either with disability scales or subjectively, time for return to play (RTP), re-injury rate, and ultrasonographic evaluation. The exclusion criteria were literature reviews, systematic reviews, studies in animals, studies in other languages, studies that failed to meet the targeted population or intervention and studies that didn't report any of the outcomes of interest. The quality of the studies was analyzed using the Cochrane Assessment Tool, the Newcastle-Ottawa Quality Assessment Scale, and the JBI Critical Appraisal Checklist. Eight studies were included in the systematic review (two randomized controlled trials, one prospective observational study, two retrospective observational studies, and three case reports), with a total of 143 adult participants. ESWT was associated with less pain on VAS, better function, reduction of size of lesion on ultrasound evaluation, faster RTP and/or lower re-injury rate in patients with indirect and direct muscle injuries and muscular hematomas, a frequent secondary complication of muscle injuries. The evidence regarding the use of ESWT for these types of injuries is therefore promising. Nevertheless, higher-quality studies are needed in the future to prove its efficacy, better comprehend its mechanisms of action and define treatment protocols (timing, type and parameters of ESWT).

## Introduction and background

Muscle injuries commonly occur in sports and are associated with significant financial burden [1], constituting 46% of American football injuries [2], 31% of soccer/football injuries [3], 17.7% of basketball injuries [4], and 10.4% of rugby injuries [5]. The muscles that are usually involved are bi-articular [6] and undergo excessive eccentric contraction [7], such as an acute hamstring muscle injury at the terminal stage of the swing phase of running. The high relevance of muscle injuries is demonstrated by the fact that an elite-level soccer team with a squad of 25 players can expect about 15 muscle injuries each season with a mean absence time of 233 days, 148 missed training sessions, and 37 missed matches [3].

Since muscles exist in different sizes and shapes with complex functional and anatomical organization, muscle injuries have traditionally been difficult to define and categorize, and different classification systems have been published in the literature throughout the years [8-11]. In an attempt to improve communication and comparability, the Munich Consensus Statement (MCS) was published in 2013 [12], which presented standardized terminology as well as a comprehensive classification for athletic muscle injuries. According to this consensus, muscle injuries can be classified into indirect muscle injuries (including functional and structural injuries) and direct muscle injuries (including contusions and lacerations). In addition, muscle injuries with tendinous involvement are either consistent with a partial or total tear in this classification system and can be included in that aspect of the classification [12]. Despite of this, scientific data regarding the prognostic and therapeutic implications of this classification are still missing.

Nowadays, extracorporeal shock wave therapy (ESWT), which includes focal and radial shock waves, is used for numerous clinical indications such as calcific tendonitis of the shoulder, Achilles tendinopathy, and plantar fasciitis [13], but to our knowledge, no systematic review evaluating the use of ESWT in the treatment of muscle injuries has been published to date. Focal and radial shock waves differ not only in their physical characteristics and in the technique for generating them, but also in the order of magnitude of the parameters usually used and in the therapeutic penetration depths into the tissue. Interestingly, the stimulation effects and therapeutic mechanism seem to be partly similar, despite the physical differences and the resulting different application areas (on the surface and depth) [14].

The treatment of most muscle injuries is conservative and based on a progressive, multimodal therapy program that includes the PRICE (Protection, Rest, Ice and Elevation) and POLICE (Protection, Optimal Loading, Ice, Compression and Elevation) protocols, passive and active stretching, physical therapies, functional rehabilitation, and general athletic reconditioning [15]. Since recent evidence suggests that ESWT improves muscular microcirculation [16] and may increase regeneration after acute muscle injury [17], one could debate whether ESWT could be integrated as part of this multimodal approach. By performing a systematic review of contemporary literature, we aim to assess the efficacy and safety of ESWT in the treatment of muscle injuries.

## Review

Methods

Search Strategy

The present systematic review was conducted according to the recommendations of the Preferred Reporting Items for Systematic Reviews and Meta-Analyses (PRISMA) statement. The following electronic databases were searched: Medline (PubMed) and Cochrane Library, without limitation of year of publication or journal, using the following keywords: (“extracorporeal shock wave therapy” OR “shock wave therapy” OR “ESWT”) AND (“muscle injury” OR “muscle injuries” OR “muscle tear” OR “muscle tears” OR “tear” OR delayed onset muscle soreness “DOMS” OR “contusion” OR “hematoma” OR “laceration”). The literature search included articles from May 1987 to June 2023.

Inclusion Criteria

Case controls, retrospective studies, prospective studies, or randomized controlled studies in English, Portuguese, and Spanish were included. The targeted population of the present systematic review was individuals aged ≥18 years old with indirect and direct muscle injuries treated with ESWT. The included studies needed to report at least one of the following outcomes: pain on visual analog scale, functionality assessed either with disability scales or subjectively, time for return to play, re-injury rate, and ultrasonographic evaluation, without limitation of minimum follow-up duration.

Exclusion Criteria

Literature reviews, systematic reviews; studies in animals, as well as studies in other languages were excluded. Studies that failed to meet the targeted population or intervention and studies that didn't report any of the outcomes of interest were also excluded.

Data Extraction

The abstracts of retrieved studies were independently reviewed by two authors, and full articles were examined when necessary. The data was extracted independently by the two authors, and any disagreements were resolved by discussion with at least one more author until a consensus was reached. 

Quality Assessment

Randomized controlled trials (RCTs) were analyzed using the Cochrane Assessment Tool, while prospective and retrospective observational studies were assessed for the risk of bias using the Newcastle-Ottawa Quality Assessment Scale. We also assessed the included case reports using the JBI Critical Appraisal Checklist. 

Results

**Figure 1:**
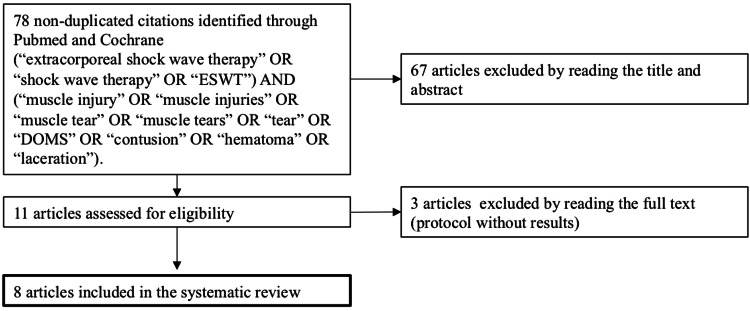
PRISMA flowchart diagram. PRISMA: Preferred Reporting Items for Systematic Reviews and Meta-Analyses; ESWT: extracorporeal shock wave therapy; DOMS: delayed onset muscle soreness.

Initially, 78 potentially relevant non-duplicated articles were screened, and 67 articles were excluded after reading the title and abstract, in accordance with the defined inclusion and exclusion criteria. Of the 11 articles assessed by reading the full text, three articles were excluded by reading the full text, and eight articles were included in the systematic review (Figure 1). These comprise two randomized controlled trials [18,19], one prospective observational study [20], two retrospective observational studies [21,22], and three case reports [23-25].

A total of 143 adult participants were included in our systematic review, of which 98 performed radial or focal ESWT. They were divided based on their injury type according to the 2013 Munich Consensus Statement (Tables 1-4). Although only two of the retrieved studies used this classification system [18,21], we reclassified all lesions in accordance to the Munich Consensus based on the mechanism of injury and/or severity of lesion for a better systematization of the published literature. In addition, despite three studies being related to ESWT in muscle injuries with tendinous involvement [19,22,23], these were also included in our systematic review since, according to Mueller-Wolfart et al., tendinous injuries can be included in this classification system [12]. Finally, in two of the retrieved studies [24,25], although a muscle injury was the primary event, ESWT was used to treat a muscular hematoma.

**Table 1:** ESWT in muscle injuries: study characteristics. ESWT: extracorporeal shock wave therapy, DOMS: delayed onset muscle soreness.

Type of muscle injury according to the Munich Consensus Statement	Study	Sample size (n)	Age (mean ± SD)	Study population	Intervention	Timing of ESWT	Type and parameters of ESWT
Indirect, type 1A	Morgan et al. 2021 (retrospective study) [21]	Eight males	18-35 years old	Professional football players with type 1A muscle-injury	Customized multimodal therapy approach including ESWT	Starting on D0 after injury (when possible)	Radial; one to six sessions; one day interval; 1.0-3.5 bar according to tolerance; 20 Hz; 6000-12000 pulses per session
Indirect, type 1B	Fleckenstein et al., 2016 (RCT) [18]	46 (23 female; 23 male)	29 ± 3.0	Healthy adults with induced type 1B muscle injury of the non-dominant biceps brachialis muscle	Group 1 (n=16): received ESWT; group 2 (n=15): received sham shock wave; group 3 (n=15): did not receive treatment (control group)	Immediately after the induction of DOMS	Focal; one session; 0.06-0.09 mJ/mm^2^; 1400 pulses total at seven equidistant points (200 pulses each)
Indirect, type 2B	Morgan et al., 2021 (retrospective study) [21]	Five males	18-35 years old	Professional football players with type 2B muscle-injury	Customized multimodal therapy approach including ESWT	Starting on D0 after injury (when possible)	Radial; 1 to 12 sessions, mostly one-day interval; 1.0-3.5 bar according to tolerance; 20 Hz; 6000-12000 pulses
Indirect, type 3A	Morgan et al., 2021 (retrospective study) [21]	Four males	18-35 years old	Professional football players with type 3A muscle-injury	Customized multimodal therapy approach including ESWT	Starting on D0 after injury (when possible)	Radial; 4 to 11 sessions, intervals mostly one day; 1.0-3.5 bar according to tolerance; 20 Hz; 6000-12000 pulses per session
Indirect, type 3B	Astur et al., 2015 (prospective study) [20]	Eight (six males; two females)	29.1 years (range, 19-51 years old)	Seven athletes with grade II muscle injury and one athlete with grade III muscle injury	Physical therapy protocol lasting four to six weeks, including ESWT	Average injury time of 8.75 weeks	Focal; one to two sessions; three-week interval; 0.03-0.06 mJ/mm^2^; 4 Hz; 900 pulses per session
Indirect, type 4	Fuentes et al., 2019 (case report) [24]	One male	53 years old	Grade III tear of the plantaris muscle complicated by hematoma	ESWT	20 Days after injury	Focal; three sessions; one-week interval; 0.040 mJ/mm^2^, 3000 pulses (500 pulses per point 2 cm apart)
Direct, contusion	Morgan et al., 2021 (retrospective study) [21]	Three males	18-35 years old	Professional football players with contusion muscle injury	Customized multimodal therapy approach including ESWT	Starting on D0 after injury (when possible)	Radial; two to three sessions; one-day interval, 1.0-3.5 bar according to tolerance; 20 Hz; 6000-12000 pulses per session; note: 1 athlete received focal ESWT
	Jung et al., 2020 (case report) [25]	One male	65-year-old man	Previously healthy adult with a painful hematoma in the left calf due to trauma	ESWT	Two weeks after the injury	Focal; one session; 0.056 mJ/mm^2^ (gradual intensity increase); 6 Hz; 3000 pulses

**Table 2:** ESWT in muscle injuries: outcomes. *Indicates that although a muscle injury was the primary event, ESWT was used to treat a muscular hematoma, a frequent complication of muscular injuries. ESWT: extracorporeal shock wave therapy, VAS: visual analog scale.

Type of muscle injury according to the Munich Consensus Statement	Study	Follow-up timeline	Pain according to VAS (mean ± SD)	Functionality assessment	Sonographic evaluation	Time for RTP (mean)	Re-injury rate
Indirect, type 1A	Morgan et al., 2021 [21]	T0-day of injury; T1-day/previous day of return-to-play; T2- two months	4.6 at T0; 0.9 at T1	NA	NA	3.3 days	12.5% within two months after RTP (T2)
Indirect, type 1B	Fleckenstein et al., 2016 [18]	T0-immediately after induction of DOMS; T1-at 72 h	ESWT group: 1.8 ± 2.3 at T0; 2.6 ± 2.9 at T1; Sham group: 2.3 ± 2.1 at T0; 4.5 ± 2.3 at T1; control group: 2.3 ± 3.0 at T0; 5.0 ± 3.1 at T1 (p=0.094)	ESWT group: 1.5 at T0; 1.8 at T1; Sham group: 1.7 ± 1.4 at T0; 3.0 ± 2.2 at T1; control group: 2.1 at T0; 3.5 at T1 (p=0.248); Note: values correspond to modified Morrey-score	NA	NA	NA
Indirect, type 2B	Morgan et al., 2021 [21]	T0-day of injury; T1-day/previous day of return-to-play; T2-two months	4.2 at T0; 1.2 at T1	NA	NA	6.2 days	0% within two months after RTP (T2)
Indirect, type 3A	Morgan et al., 2021 [21]	T0-day of injury; T1-day /previous day of return-to-play; T2-two months	5 at T0; 1.25 at T1	NA	NA	13 days	0% within two months after RTP (T2)
Indirect, type 3B	Astur et al., 2015 [20]	T0-before the start of treatment; T1-six weeks of treatment	5.75 at T0; 0.5 at T1	Eight at T0; six at T1; Note: values correspond to Tegner Scale	4.2 × 2.2 × 2.1 cm at T0; 2.2 × 1.3 × 1.1 cm at T1	Eight weeks	NA
Indirect, type 4	Fuentes et al., 2019* [24]	T0-day of injury; T1-day of first sonographic evaluation (seven days); T2- first session of ESWT (20 days); T3-third session of ESWT (51 days); T4-last day of follow up (53 days)	Nine at T2; two at T3; 0 at T4	Gate with crutches at T0; no need of crutches at T3; completely functional at T4	9 × 3 cm in longitudinal axis; 5 × 2 cm in transversal axis at T1; 3.3 × 1 cm in longitudinal axis; 2.5 × 1 cm in transversal axis at T4	NA	NA
Direct contusion	Morgan et al., 2021 [21]	T0-day of injury; T1-day/previous day of return-to-play; T2-two months	4.2 at T0; 1 at T1	NA	NA	Four days	0% within two months after RTP (T2)
	Jung et al., 2020* [25]	T0-immediately before ESWT; T1-immediately after ESWT; T2-two weeks	Seven at T0; three at T1; 0 at T2	Gate with crutches at T0; gate without crutches at T1	4.3 cm × 1.5 cm × 4.9 cm at T0; 4.2 cm × 1.4 cm × 4.6 cm at T1	NA	NA

**Table 3:** ESWT in tendinous injuries: study characteristics. ESWT: extracorporeal shock wave therapy, NAG: non-athletic group.

Type of tendinous injury according to the Munich Consensus Statement	Study	Sample size (n)	Age (mean ± SD)	Study population	Intervention	Timing of ESWT	Type of ESWT and parameters
Indirect, type 3	Chou et al., 2018 (retrospective study) [22]	36	26.6 ± 5.6 in AG; 52.7 ± 10.3 in NAG	Thirteen professional athletes and 23 non-athletes with shoulder tendinitis or partial tears of the rotator cuff (mostly grade I tears according to Ellman)	One session of focal ESWT	≥3 Months of injury time	Focal; one session; 0.28-0.3 mm/mJ^2^; frequency not specified; 3000 impulses
	Notarnicola et al., 2020 (RCT) [19]	30	65.2 ± 8.7 years	Adults with partial tears of the supraspinatus tendon (mostly grade III tears according to Ellman)	ESWT group (n=15): three sessions of focal ESWT; exercise group (n=15): 12-week physical therapy program	Average symptom duration of 12.6 ± 8.2 months	Focal; three sessions, one week interval, 0.05 mJ/mm^2^; frequency not specified; 2000 impulses per session
	Hsu et al., 2017 (case report) [23]	One female	64-year-old	Partial tear of Achilles tendon	Physical therapy program including focal ESWT	Six months after the injury	Focal; eight sessions; two weeks apart (last five sessions one week apart); 0.142-0.341 mJ/mm^2^; 6 Hz; 2500 impulses per session (first 500 pulses applied at soleus muscle)

**Table 4:** ESWT in tendinous injuries: outcomes. ESWT: extracorporeal shock wave therapy; VAS: visual analog scale; ADL: activities of daily life.

Type of tendinous injury according to the Munich Consensus Statement	Study	Follow-up	Pain according to VAS (mean ± SD)	Functionality assessment	Sonographic evaluation	Time for RTP	Re-injury rate
Indirect, type 3	Chou et al., 2018 [22]	T0-initial; T1-three months; T2-six months; T3-12 months	AG: 5.1 ± 0.9 at T0; 3.8 ± 1.5 at T1 2.0 ± 2.8 at T3; NAG: 5.6 ± 0.8 at T0; 4.3 ± 1.6 at T1; 2.6 ± 2.9 at T3	AG: 57.9 ± 12.9 at T0; 69.2 ± 10.1 at T1; 79.0 ± 10.5 at T2; 86.8 ± 12.3 at T3; NAG: 52.7 ± 14.2 at T0; 62.5 ± 14.0 at T1; 71.1 ± 15.7 at T2; 78.8 ± 18.3 at T3; Note: values correspond to the constant score	NA	15% of players in the athletic group return to the previous competitive level within three months after ESWT	62% in AG and 18% in NAG at T3
	Notarnicola et al., 2020 [19]	T0: initial; T1: three months	ESWT group: 7.4 ± 1.4 at T0; 3.9 ± 2.2 at T1; exercise group: 7.5 ± 0.8 at T0; 6.9 ± 1.2 at T1 (p=0.013)	NA	ESWT group: 6.3 ± 1.5 × 5.2 ± 1.5 × 2.9 ± 0.8 mm at T0; 5.3 ± 2.4 × 4.1 ± 2.1 × 1.9 ± 0.9 mm at T1; Exercise group: 6.4 ± 1 × 5.5 ± 18 × 2.09 ± 8 mm at T0; 6.2 ± 1.8 × 5.4 ± 3.4 × 2.5 ± 1.0 mm at T1 (p=0.049)	NA	NA
	Hsu et al., 2017 [23]	T0-initial; T1-three months; T2-four months	Nine at T0; one at T1	Dependent in ADL at T0; independent in ADL at T1	Multiple partial tears 1 cm proximal to calcaneus at T1; fiber filament noted in the previous hypoechoic lesions at T2	NA	NA

Pain

Pain according to VAS was reported by all authors [18-25]. In indirect type 1A, type 2B, type 3A, and direct contusion muscle injuries, Morgan et al. reported a pain reduction of approximately 3.7 points, 3 points, 3.75 points, and 3.2 points, respectively, compared to baseline at the day or previous day of return-to-play [21]. In type 1B muscle injuries, Fleckenstein et al. reported a non-significant reduction in pain compared to sham and control (p=0.094) but significant when compared to the control group alone (p=0.041), at 72 h follow-up [18]. In type 3B muscle injuries, Astur et al. reported a pain reduction of approximately 5.3 points compared to baseline at six weeks of follow-up [20]. In tendinous injuries, all authors reported pain reduction after ESWT at three months [19,22,23], with special emphasis on Notarnicola et al. who reported a statistically significant difference in the interaction between time and group (p=0.013) [19]. When treating secondary muscular hematomas, Fuentes et al. reported a pain reduction of seven points compared to baseline three weeks after the beginning of treatment [24]. Similarly, Jung et al. reported a pain reduction of four points compared to baseline immediately after the application of ESWT [25]. 

Functionality

Functionality was assessed in six articles, either with disability scales (modified Morrey-Score, Tegner Scale, and Constant Score) or subjectively [18,20,22-25]. Although Morgan et al. didn't assess their athletes functionality, we can assume that all of them were completely fit at return-to-play [21] (see below). In type 1B muscle injuries, Fleckenstein et al. reported that the ESWT didn't have a statistically significant difference compared to the sham and control groups or the control group only at 72 h follow-up (p=0.248 and p=0.103) [18]. In type 3B muscle injuries, Astur et al. reported a reduction of approximately two points from baseline in the Tegner Scale, at six weeks of follow-up [20]. In tendinous injuries, Chou et al. reported an increase from baseline of approximately 29 points and 26 points in the Constant Score in the athletic group and non-athletic group, respectively, at the 12-month follow-up. In addition, 53.8% of participants in the athletic group were significantly better at 12-month follow-up, returning to their previous level of activity [22]. In secondary hematomas, Fuentes et al. subjectively reported that the patient didn't need crutches for walking after three sessions of ESWT [24]. Similarly, Jung et al. also reported that the patient could walk without crutches immediately after ESWT [25].

Sonographic Evaluation

Lesions were assessed with ultrasound in five articles [19,20,23-25]. In type 3B muscle injuries, Astur et al. reported a decrease of lesion size from 4.2 × 2.2 × 2.1 cm to 2.2 × 1-3 × 1.1 cm at six weeks follow-up [20]. In tendinous lesions, Notarnicola et al. reported a significant difference in the interaction between time and group for the size of the lesion on the antero-posterior axis (p=0.049) [19]. Similarly, Hsu et al. reported the existence of newly formed fiber filaments in the previous hypoechoic lesions at a four-month follow-up [23]. In secondary muscular hematomas, Fuentes et al. reported that the hematoma decreased from 9 × 3 cm in the longitudinal axis and 5 × 2 cm in the transversal axis to 3.3 × 1 cm in the longitudinal axis and 2.5 × 1 cm in the transversal axis after ESWT [24]. Jung et al. also reported a decrease in the size of the hematoma secondary to the lesion from 4.3 × 1.5 × 4.9 cm to 4.2 × 1.4 × 4.6 cm immediately after ESWT [25].

Time for Return to play

Only three articles reported time for RTP [20-22]. Morgan et al. reported that the time to return to play was 3.3 days for type 1A muscle injuries, 6.2 days for type 2B muscle injuries, 13 days for type 3A muscle injuries, and four days for contusions [21]. Astur et al. reported a mean time for RTP of eight weeks in type 3B muscle injuries [20]. Chou et al. reported that 15% of patients in the athletic group with type 3 tendinous injuries return to their previous competition level within three months of ESWT [22].

Re-injury rate

Only two articles reported the re-injury rate [21,22]. Morgan et al. reported that the re-injury rate within two months was 12.5 % in type 1A muscle injuries and 0% in type 2B, type 3A, and contusion muscle injuries [21]. Chou et al. reported a re-injury rate of 62% in tendinous injuries in the athletic group and 18% in the non-athletic group at 12-month follow-up [22].

Discussion

Muscle Injuries

As reported by Morgan et al. [21], integrating radial ESWT into a multimodal therapy approach shortened mean lay-off time by 54% in type 1A muscle injuries, 50% in type 2B muscle injuries, and 8% in type 3B muscle injuries, compared to the data reported by Erkstrand et al [26]. In addition, the toral re-injury rates were 4% in functional/ultrastructural injuries and 0% in type 3A muscle injuries, compared to 12% and 13%, respectively, in the study by Erkstrand et al [26]. Based on these results, ESWT may not only help to reduce layoff times but could also help in the prevention of muscle re-injury among athletes with these types of injuries. In the RCT published by Fleckenstein et al., the effects of focal ESWT on pain after induction of a type 1B muscle injury were negligible during the first 24 h. However, after 72 h, focal EWST significantly reduced pain and impairment (p<0.094 and p<0.248, respectively). The results of this study need to be carefully interpreted since the results were adjusted for multiple comparisons, which lead to the study being underpowered. For instance, when compared to control only, pain and impairment were reduced by almost 50% (p=0.041 and p=0.103, respectively) [18]. As a result, EWST appears to promote a quicker recovery of previous performance levels (e.g., enhancing athletes return to play) rather than being another analgesic modality in the treatment of DOMS [27]. Astur et al. treated athletes with chronic type 3B muscle injuries (>3 weeks), which are injuries even more difficult to treat than acute injuries because of the associated fibrosis and hematoma remains. Nevertheless, the improvement of patients with focal ESWT associated with physiotherapy was quite significant, with all athletes achieving a return to play approximately eight weeks after the beginning of treatment [20]. Although these are very different types of muscle injuries and the exact mechanisms that may have contributed to outcomes are not well established, these could be related to the molecular and cellular effects of ESWT, which include analgesia [28], muscular relaxation [29], neovascularization [30], increased muscular microcirculation [16], and stimulation of regeneration of skeletal muscle tissue and acceleration of repair processes [17].

Tendinous Injuries

The reported success rate of arthroscopic debridement of partial tears of the rotator cuff in competitive throwing athletes ranges from 50% to 85%, and the duration of a return to the previous activity level is usually longer than six months [31,32]. ESWT has been proven to have a trophic and reparative effect on tendon tissue [33]. In the retrospective study published by Chou et al. [22], 53.8% of the subjects in the AG had a high satisfaction rate with focal ESWT at 12 months, returning to their previous level of activity, which was similar to the reported outcome of surgical intervention. Notarnicola et al. also reported that focal ESWT allowed us to obtain both improvement and reduction in the size of the tendon lesion (on the antero-posterior axis), with statistical significance [19]. Hsu et al. reported a case of a partial Achilles tendon tear successfully treated with focal ESWT [23]. Therefore, ESWT could be an appropriate alternative treatment after the failure of other conservative treatments, owing to the non-invasive nature of the treatment, before considering surgery.

Complications of Muscle Injuries

Muscle hematomas can be the consequence of an external blunt (direct trauma) or of an excessive or uncoordinated contraction (indirect trauma) [34]. It is known that these lesions can lead to the formation of scar tissue, further complicating the healing process [35]. Fuentes et al. described a case report of successful treatment of a subacute hematoma secondary to a type 4 lesion of the plantaris muscle using focal ESWT [24]. Similarly, Jung et al. reported another case of a patient with a subacute hematoma of the gastrocnemius following contusion that was successfully treated with focal ESWT [25]. Although the mechanism of action of ESWT on muscle hematomas is unknown, as reviewed in Zissler et al., ESWT could reduce a chronic condition to an acute response [17], leading to a quicker resorption of the hematoma. Besides, ESWT leads to a decrease in capsular fibrosis associated with silicone devices related with synergic alterations in pro- and anti-fibrotic proteins (transforming growth factor ß1 and matrix metalloproteinase 2, respectively) [36]. Therefore, EWST may also affect capsule breakdown and deposit disorganization in unabsorbable hematomas with fibrous capsules, as described by Jung et al [25]. Although these are only case reports, ESWT could be a minimally invasive alternative for the treatment of painful hematomas.

Timing, Type and Parameters of ESWT 

The timing of application of ESWT after injury, the type of ESWT used (radial or focal), and the parameters (number of sessions, interval between sessions, energy flux density (EFD) or pressure, frequency, impulses per session) are extremely relevant aspects in the treatment of muscle and tendinous injuries since it is usually a progressive and multimodal process [15]. Based on the results obtained by Morgan et al. and Fleckenstein et al. [18,21], we hypothesize that radial or focal ESWT applied immediately after functional muscle injury could be beneficial due to the already-mentioned effects that ESWT has on pain and muscular tonus. On the other hand, when using ESWT for structural muscle or tendinous injuries, it could be prudent to limit its use to the fibroblastic and remodeling phases of tissue healing [37] with preferentially lower energy of flux density/pressure since (1) we don’t want to further aggravate the lesion and (2) lower and medium EFD trigger the release of nitric oxide, which is beneficial to its antalgic, angiogenic, and anti-inflammatory effects [38].

Strengths and Limitations

The use of ESWT is well documented in medical literature for the treatment of conditions such as calcific tendonitis of the shoulder, Achilles tendinopathy, and plantar fasciitis. To our knowledge, this systematic review is the first one to evaluate the efficacy and safety of ESWT in the treatment of muscle injuries, and although the results are promising, most published studies are observational studies without control groups and heterogeneous in the reported outcomes. In addition, muscle injuries are very different in pathophysiology, and consequently, treatment and prognosis might vary. Therefore, future higher-quality studies are needed to better comprehend the mechanisms of action of ESWT depending on injury type and also define protocols regarding timing, type and parameters of ESWT for optimized treatment.

## Conclusions

ESWT appears to be effective in the treatment of patients with indirect and direct muscle injuries and muscular hematomas, a frequent secondary complication of muscle injuries. Nevertheless, future higher-quality studies are needed to prove its efficacy and safety, better comprehend its mechanisms of action, and define protocols (timing, type and parameters of ESWT) depending on the type of injury.
